# Effect of sarcopenia and poor balance on vertebral spinal osteoporotic fracture in female rheumatoid arthritis

**DOI:** 10.1038/s41598-022-13339-2

**Published:** 2022-06-08

**Authors:** Mei Zhang, Shengqian Xu, Hexiang Zong, Jianxiong Wang, Yiran Chu, Jingyu Cai, Ling Chang

**Affiliations:** 1 Department of Rheumatology and Nephrology, The Fourth Affiliated Hospital of Anhui Medical University, Hefei, 230000 China; 2 Department of Rheumatology and Immunology, The First Affiliated Hospital of Anhui Medical University, Hefei, 230022 China

**Keywords:** Rheumatology, Risk factors

## Abstract

This study investigated the effect of poor balance and sarcopenia on vertebral spinal osteoporotic fracture (VOPF) in female rheumatoid arthritic (RA) patients. A total of 195 female RA and 126 normal subjects were enrolled, and the correlations between sarcopenia, poor balance and VOPF were analyzed. Furthermore, we explored the relationships between sarcopenia or poor balance with disease related indexes of female RA. Binary logistic regression analyses were performed to identify potential risk factors for VOPF in female RA. We found that female RA had an increased risk of sarcopenia, poor balance (Berg balance scale, BBS ≤ 40) and VOPF than controls (*P* < 0.0001). Female RA with VOPF were more likely to have poor balance and sarcopenia than those without VOPF (*P* < 0.0001–0.05). Meanwhile, female RA with sarcopenia and poor balance often had higher disease activity, more serious joint damage and worse joint function (*P* < 0.05) compared with those without sarcopenia and poor balance. Binary logistic regression analysis (LR backwald) revealed that age (OR = 1.112, 95% CI 1.065–1.160, *P* < 0.0001), OP (OR = 10.137, 95% CI 4.224–24.330, *P* < 0.0001) and GCs usage (OR = 3.532, 95% CI 1.427–8.741, *P* = 0.006) were risk factors, while SMI (OR = 0.386, 95% CI 0.243–0.614, *P* < 0.0001) and BBS (OR = 0.952, 95% CI 0.929–0.976, *P* < 0.0001) were protective factors for VOPF in female RA. Hence, sarcopenia and poor balance are associated with a higher risk for VOPF and are closely related to disease activity and joint structure damage of female RA.

## Introduction

Rheumatoid arthritis (RA) is a common autoimmune and inflammatory disease. Its inflammatory condition predisposed patients to induce the development of osteoporosis (OP). OP in RA has a multifactorial pathogenesis with systemic inflammation, and glucocorticoid use plays major roles^1^. Typical symptoms of RA include local and systemic bone loss in the joints, and the latter leads to OP and an increased risk of fragility fractures^1,2^. The latter is also known as osteoporotic fracture (OPF), which further impairs functional ability, quality of life, and life expectancy^3^. OPF can occur with even minor trauma or collision during daily work, and is commonly found in the vertebrae, hip, pelvis, distal forearm, and proximal humerus, with the vertebrae being the most common^4^. Comprehensive domestic and international studies had shown that the occurrence of OP in RA patients was about 30–50%, which was significantly higher than that in the normal population^5,6^, while the risk of OPF in RA was 2–5 times higher than that in the normal population^7^. Two meta-analyses showed a 60–100% higher risk of fracture in RA patients compared to healthy controls^8,9^. Another meta-analysis, focused only on vertebral fractures, confirmed a similar two-fold increase in vertebral fracture risk in RA patients^10^. RA existed more common in women than men (3:1), its disease activity seemed to be higher in women. Therefore, OP, one of comorbidities in RA, is more frequent in females^11^.

Sarcopenia leds to age-associated muscle myopathy, characterized by a generalized progressive disease of reduced muscle mass and strength resulting in disability, lower quality of life and even fatal symptom^12^. It had been reported that the prevalence of sarcopenia in RA patients was about 25.9–43.3%^12,13^, and sarcopenia was associated with the development of OP and might be a risk factor for OPF. The combined effect of sarcopenia and OP could lead to moving difficulties, falling, and fragility fractures, which could be catastrophic for the seniors^14^.

Furthermore, with joint and ligament damage and decreased muscle strength, RA patients often sway during standing and walking, have difficulty in posture controlling. The risk of falling and fractures in RA patients increased frequently is due to poor balance capacity.

Since the risk of VOPF is significantly higher in female RA than those in normal people, it is essential for us to find new risk factors related to RA. In our study, we investigated the role of sarcopenia and balance ability in the occurrence of VOPF in female RA patients, thereby provide a theoretical foundation for the clinical prevention and treatment of VOPF in female RA patients.

## Methods

### Clinical data

195 female RA patients attending the Department of Rheumatology and Immunology of the First Affiliated Hospital of Anhui Medical University from January 2016 to January 2020 were enrolled, all of whom fulfilled the diagnostic criteria for RA classification proposed by the ACR in 1987 and the latest diagnostic criteria for RA proposed at the ACR meeting in 2009. 126 age-and gender-matched healthy individuals from Medical Examination Center of the First Affiliated Hospital of Anhui Medical University were also selected as controls. The number of subjects needed to be enrolled in the study is calculated with an alpha of 0.05 and 80% power for hypothesis tests. RA patients were asked about disease duration and previous medication use, including glucocorticoids (GCs). 110 (56.4%) patients used GCs in the disease duration and the average daily dose was 4.83 ± 5.29 mg. 81 (41.54%) patients used conventional synthetic disease-modifying antirheumatic drugs (csDMARDs). The exclusion criteria included patients with an acute or chronic infectious disease such as thyroid diseases or parathyroid diseases, other endocrinal diseases, severe liver, kidney disease, or primary hematological disorders. If estrogen, androgen, anticonvulsant, anticoagulant, or any kinds of anti-anemia drugs, such as chalybeates or folic acid, were used simultaneously, the patient was also excluded. Other exclusion criteria were alcoholics, smokers, and HIV subjects. Patients who were pregnant or breastfeeding and patients with a history of other inflammatory or non-inflammatory arthritis, severe trauma, infectious and inflammatory diseases, and other decompensated diseases were excluded from the study. The study was conducted in accordance with the principles of the Declaration of Helsinki, and all the individuals enrolled were informed about the objectives of the study and gave their consent. The Ethics Committee of Anhui Medical University approved the study (approval number: 20121090).

### Clinical data recording and testing methods

#### Clinical data recording and assessment

All subjects' age, gender, height, weight, disease duration, joint tenderness count (JTC) and joint swelling count (JSC) were recorded in details, and body mass index (BMI) was calculated by dividing weight by height squared (kg/m^2^). Standard laboratory tests were performed to measure hemoglobin (Hb), erythrocyte sedimentation rate (ESR), C-reactive protein (CRP), rheumatoid factor (RF), and anti-cyclic peptide containing citrulline (anti-CCP) in the peripheral blood of female RA. The disease activity of female RA was evaluated by calculating DAS28. The joint function of female RA patients were determined by revised criteria for the classification of global functional status (from grade I to grade IV), referring to the ACR 1991^15^. The health assessment questionnaire (HAQ) was used to assess the daily living ability of female RA patients. The HAQ score ranged from 0 to 3, with higher scores indicating higher degree of disability.

#### Muscle mass measurement and sarcopenia judgment criteria

Bioelectrical impedance analysis (BIA) (InBody 720: BioSpace Co., Ltd., Seoul, Korea) was used to estimate muscle mass based on the difference in water and electrolyte content between skeletal muscle and adipose tissue. The assessment involved test on skeletal muscle mass of five segment of body: the trunk, the left and right arms, the left and right legs. Skeletal muscle mass index (SMI) was calculated by the sum of an arm and lean leg mass (kg)/height (m) squared. The diagnostic criteria for sarcopenia established by the Asian Working Group on Skeletal Dyskinesia included the following three items: (1) SMI less than 7.0 kg/m^2^ for males and less than 5.7 kg/m^2^ for females by bioresistance method; (2) grip strength: less than 26 kg for males and less than 18 kg for females; (3) daily gait speed less than 0.8 m/s. Satisfying one of the first, and last two items was diagnosed as sarcopenia^16^. In our study, female RA patients whose SMI index lower than 5.7 kg/m^2^ was identified as sarcopenia. And female RA was divided into two groups: sarcopenia group and non-sarcopenia group.

#### Balance ability measurement and balance ability judgment criteria

The Berg balance scale (BBS) was used to assess the patient's ability to maintain balance in different postures, transitions, and predictive postural control, and was now widely used in many rehabilitation settings^17^. It used a 5-point sequential scale to evaluate participants performing 14 functional activities such as standing up, sitting down, standing independently, standing with eyes closed, upper arm extension, turning around, alternating steps with both feet, and standing on single leg. Each item was scored 0–4, with a total score of 56^18^. Higher scores indicated better balance, while lower scores indicated poorer balance and higher risk of falling. According to the BBS, female RA patients were divided into good-balance group (BBS > 40 points) and poor-balance group (BBS ≤ 40 points).

#### Bone mineral density (BMD) measurement and OP judgment criteria

BMD was measured at the femoral neck (Neck), total hip (Hip) and L1–L4 in all study subjects using a GE Lunar Prodigy dual-energy X-ray bone densitometer and expressed in g/cm^2^. According to the World Health Organization classification, osteoporosis was defined by the BMD assessment at the hip or lumbar spine, which was less than or equal to 2.5 standard deviations below the mean BMD of a young-adult reference population^19^.

#### Hand X-rays and Sharp scores

Using a MECALL Castor-50-hf X-ray scanner, radiographs of both hands (including wrists and fingers) were performed on the subjects to assess X-ray staging (grades I–IV). Joint space narrowing and bone erosion of both hands including both wrists were calculated according to the Sharp^20^, with a maximum joint space narrowing score of 144 and a maximum joint erosion score of 170 in all regions, for a total Sharp score of 0–314. Sharp score were evaluated by two independent radiologists back to back by blinded method.

#### Judgment criteria for spinal X-rays and VOPF

Genant's semi-quantitative method (semi quantity, SQ) was used as a criterion for determining VOPF^21^: vertebral body decreased by more than 20% of height in the anterior, middle and posterior and the area reduce more than 10%. Female RA patients were divided into two groups: VOPF group and non-VOPF group.

### Statistical analysis

SPSS23.0 software was used for statistical analysis. The measurement data were expressed as $\stackrel{\mathrm{-}}{\text{x}}$ ± s (normal distribution) or M (P25–P75) (non-normal distribution). *t*-test or non-parametric test was used for comparison of measurement data between two groups, *x*^*2*^ test was used for comparison of count data. We applied binary logistic regression analysis (LR backwald) to analyze the risk factors. A difference was considered statistically significant at *P* < 0.05.

## Results

### Comparison of clinical features between female RA and controls

We included 195 patients in this study. Table 1 shows the characteristics of the patients. Average disease duration was 9.56 ± 9.19 years, average BMI was 22.38 ± 3.81 kg/m^2^. 110 patients (56.4%) had used GCs and 81 patients (41.54%) had used csDMARDs. Average DAS28 was 5.48 ± 1.15. 116 patients (59.5%) were in high disease activity, 79 patients (40.5%) in low-moderate disease activity. The average SMI of 195 female RA (cases) was 5.37 (4.67–6.03) kg/m^2^, significantly lower than that of controls (8.48 (7.89–8.96) kg/m^2^) (*x*^*2*^ = 123.924, *P* < 0.0001). The average BBS of female RA was 44 (31–51), significantly lower than that of controls (52 (48–54)) (*x*^*2*^ = 46.023, *P* < 0.0001). The average BMD at all the measured sites of female RA were significantly lower than those in controls (*P* < 0.0001) (Table 1).

**Table 1:** Comparison of clinical features between female RA and controls.

Index	Cases (*n* = 195)	Controls (*n* = 126)		*x*^2^/*t*		*P*
Age (year)	55.49 ± 12.33	54.96 ± 10.74		0.397		0.692
Height (cm)	157.76 ± 5.16	157.88 ± 6.24		–2.168		0.031*
Weight (kg)	55.75 ± 10.14	58.13 ± 8.60		–0.181		0.857
disease duration (year)	9.56 ± 9.19	–		–		–
BMI (kg/m^2^)	22.38 ± 3.81	–		–		–
GCs% (n)/daily dose range in mg	56.4 (110)/4.83 ± 5.29	–		–		–
csDMARDs% (n)	41.54 (81)	–		–		–
DAS28	5.48 ± 1.15	–		–		–
Low-moderate disease activity% (n)	40.5 (79)	–		–		–
High disease activity% (n)	59.5 (116)	–		–		–
HAQ	1.24 ± 0.72	–		–		–
ESR (mm/H)	63.26 ± 29.30	–		–		–
CRP (mg/L)	42.79 ± 46.52	–		–		–
Sharp	61.10 ± 69.38	–		–		–
SMI (kg/m^2^)	5.37 (4.67–6.03)	8.48 (7.89–8.96)		123.924		< 0.0001*
BBS	44 (31–51)	52 (48–54)		46.023		< 0.0001*
Neck-BMD (g/cm^2^)	0.79 ± 0.18	0.92 ± 0.16		6.451		< 0.0001*
Hip-BMD (g/cm^2^)	0.81 ± 0.17	0.97 ± 0.15		9.101		< 0.0001*
L1-BMD (g/cm^2^)	0.89 ± 0.17	0.98 ± 0.18		6.135		< 0.0001*
L2-BMD (g/cm^2^)	0.92 ± 0.19	1.06 ± 0.20		6.135		< 0.0001*
L3-BMD (g/cm^2^)	1.00 ± 0.20	1.13 ± 0.20		5.470		< 0.0001*
L4-BMD (g/cm^2^)	1.02 ± 0.19	1.13 ± 0.20		5.090		< 0.0001*
L1-4-BMD (g/cm^2^)	0.98 ± 0.20	1.11 ± 0.20		5.780		< 0.0001*

Mean and standard deviation for the continuous variables and number of participants (n) and percentages (%) for the categorical variables.

*P value < 0.05.

### Incidence of sarcopenia, poor balance, and OP between female RA and controls

**Figure 1:**
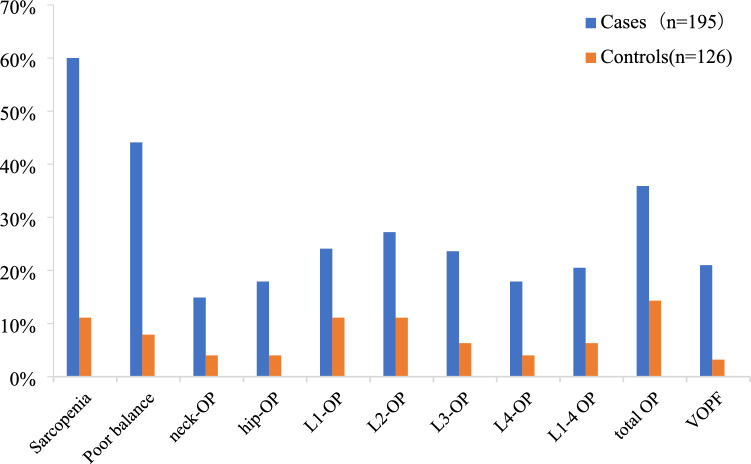
Comparison of sarcopenia, poor balance, and OP between female RA patients and controls.

The incidence of sarcopenia in 195 female RA patients (cases) was 60.0% (117/195), which was significantly higher than that in controls (11.1%, 14/126, *x*^*2*^ = 75.737, *P* < 0.0001). The prevalence of poor balance (BBS ≤ 40) in female RA patients was 44.1% (86/195), significantly higher than that in controls (7.9%, 10/126, *x*^*2*^ = 47.759, *P* < 0.0001). The prevalence of OP at Neck/Hip/L1/L2/L3/L4 in female RA patients was respectively 14.9% (29/195)/17.9% (35/195)/24.1% (47/195)/27.2% (53/195)/23.6% (46/195)/17.9% (35/195), all higher than those in controls: 4.0% (5/126)/4.0% (5/126)/11.1% (14/126)/11.1% (14/126)/6.3% (8/126)/4.0% (5/126) (*x*^*2*^ = 27.558, 48.712, 23.500, 21.393, 22.976, 21.352, *P* < 0.0001). The proportion of VOPF in female RA patients was 21.0% (41/195), significantly higher than that in controls (3.2%, 4/126, *x*^*2*^ = 20.236, *P* < 0.0001) (Fig. 1).

### Comparison of sarcopenia and poor balance between female RA with VOPF or not

**Figure 2:**
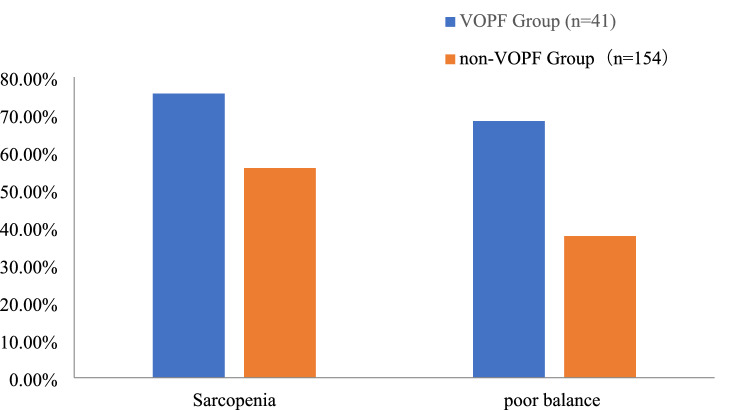
Comparison of sarcopenia and poor balance between female RA with VOPF or not.

The incidence of sarcopenia in female RA with VOPF was 75.6% (31/41), significantly higher than that in female RA without VOPF (55.8%, 86/154, *x*^*2*^ = 4.293, *P* < 0.05). The percentage of poor balance in female RA with VOPF was 68.3% (28/41), which was significantly higher than that in female RA without VOPF (37.7%, 58/154, *x*^*2*^ = 13.789, *P* < 0.0001) (Fig. 2).

### Comparison of disease-related indicators between RA with sarcopenia or not

#### Comparison of general data

**Figure 3:**
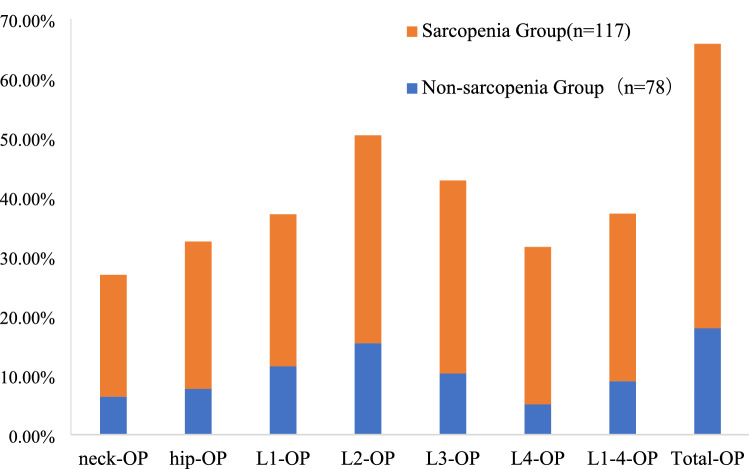
Comparison of OP between female RA patients with sarcopenia or not.

The average age in female RA patients with sarcopenia was (57.69 ± 11.51) years, which was significantly higher than that in female RA without sarcopenia (52.03 ± 12.81 years, *t* = 3.021, *P* < 0.05). Furthermore, we found that most of the sarcopenic patients were more than 60 years old (47.9%), followed by patients between 41 and 60 years (41.9%). Sarcopenia was less prevalent on patients under 40 years old. The average disease duration in female RA patients with sarcopenia was 8 (3–15) years, significantly higher than that in female RA without sarcopenia (5 (1–15) years, *z* = 2.013, *P* < 0.05). Sarcopenia group had statistically significant higher incidence of GCs usage (64.1%) compared to non-sarcopenia patients (44.9%, *x*^*2*^ = 7.039, *P* = 0.008). The average of BMI in female RA patients with sarcopenia was (21.00 ± 3.34), which was significantly lower than that in female RA without sarcopenia (24.36 ± 3.53, *t* = 6.062, *P* < 0.001) (Table 2). The percentage of neck/Hip/L1/L2/L3/L4/L1-4/total-OP in female RA with sarcopenia was significantly higher than that in female RA without sarcopenia (*x*^*2*^ = 15.277, 23.443, 15.720, 15.142, 24.889, 20.067, 19.114, 18.200, *P* < 0.001) (Fig. 3).

**Table 2:** Comparison of disease-related indexes between RA with sarcopenia or not.

Index	Non-sarcopenia (*n* = 78)	Sarcopenia (*n* = 117)	*t/z/x*^*2*^	*P*
**Age (year)**	52.03 ± 12.81	57.69 ± 11.51	3.021	< 0.05*
< 40 years% (n)	17.9 (14)	10.3 (12)		
41–60 years% (n)	56.4 (44)	41.9 (49)		
> 61 years% (n)	25.6 (20)	47.9 (56)	10.078	0.006*
Disease duration (year)	5 (1–15)	8 (3–15)	2.013	< 0.05*
GCs% (n)	44.9 (35)	64.1 (75)	7.039	0.008*
BMI (kg/m^2^)	24.36 ± 3.53	21.00 ± 3.34	6.062	< 0.01*
Joint tenderness count	6 (3–10.5)	5.6 (4–11)	0.837	0.403
Joint swelling count	10 (5–17)	12 (7–22)	1.560	0.119
VAS	5 (4–6)	5 (4–6)	0.175	0.861
ESR (mm/h)	57.78 ± 29.30	67.22 ± 28.90	2.072	0.038*
CRP (mg/l)	19.62 (6.68–49.30)	28.48 (15.50–56.87)	2.119	0.034*
RF (IU/l)	92.00 (23–187)	152.00 (38.5–201.0)	1.870	0.061
DAS28	5.30 ± 1.18	5.60 ± 1.08	1.879	0.395
Joint function (I:II:III:IV)	9:47:21:1 (11.5%:60.2%:26.9%:1.3%)	5:66:38:8 (4.2%:56.4%:32.4%:6.8%)	7.167	0.067
Joint X-ray staging (I:II:III:IV)	25:13:20:20 (32.1%:16.7%:25.6%:25.6%)	16:17:43:41 (13.7%:14.5%:36.8%:35.0%)	10.766	0.013*
HAQ	1.01 ± 0.79	1.38 ± 0.69	3.638	< 0.0001*
Sharp	10 (0.5–72.50)	53 (6.75–125.50)	3.603	< 0.0001*

Mean and standard deviation for the continuous variables and number of participants (n) and percentages (%) for the categorical variables.

*P value < 0.05.

#### Comparison of disease activity, joint function and joint structural damage between different groups

Compared with RA without sarcopenia, ESR and CRP in female RA with sarcopenia were higher, and the differences were statistically significant (*P* < 0.05–0.001), suggesting higher disease activity (Table 2). The differences in the composition of joint function grading (grades I–IV) between female RA patients with sarcopenia or not were not statistically significant (*P* = 0.067). The median HAQ score and the median Sharp score were statistically higher in the sarcopenia group than the non-sarcopenia group (*P* < 0.001). Component ratios of joint X-ray staging (grades I–IV) were significant different among female RA patients with sarcopenia or not, percentages of grade III and IV were higher in sarcopenia group than that in non-sarcopenia group (36.8% vs. 35.0% and 25.6% vs. 25.6%) (*x*^*2*^ = 10.766, *P* = 0.013)., these results suggest that RA with sarcopenia have more joint damage.

### Comparison of disease-related indicators among RA with different balance capacity

#### Comparison of general data

The average age of female RA with poor balance was 58.07 years, which was greater than that of RA with good balance (53.5 years, *P* < 0.01). The average disease duration in female RA with poor balance was 10 years, which was significantly higher than that in female RA with good balance (4.5 years, *P* < 0.0001). Poor balance patients had statistically significant higher incidence of GCs usage (65.1%) compared to good balance patients (49.5%, *x*^*2*^ = 4.742, *P* = 0.029). There was no statistically significant difference in BMI between RA with poor balance or good balance (*P* > 0.05). The percentage of total OP of RA with poor balance (47.7%) was significantly higher than that in RA with good balance (26.6%, *P* < 0.05) (Table 3).

**Table 3:** Comparison of disease related indexes between female RA with different balance capacity.

Index	Poor-balance group (BBS ≤ 40, *n* = 86)	Good-balance group (BBS > 40, *n* = 109)	*t/z/x*^*2*^	*P*
Age (year)	58.07 ± 11.332	53.36 ± 12.736	2.821	0.005*
Disease duration (year)	10.00 (5.0–20.0)	4.50 (0.92–10.75)	4.393	< 0.0001*
GCs% (n)	65.1% (56)	49.5% (54)	4.742	0.029*
BMI (kg/m^2^)	22.55 ± 3.92	22.20 ± 3.69	0.479	0.632
Total OP	41 (47.7%)	29 (26.6%)	11.485	0.003*
Joint swelling count	8.00 (4–12)	6 (3–10)	2.132	0.033*
Joint tenderness count	12.0 (7–25)	9 (5–16)	3.115	0.002*
VAS	5 (5–7)	5 (4–6)	4.151	< 0.0001*
ESR (mm/h)	70.43 ± 27.72	58.01 ± 29.57	2.964	0.003*
CRP (mg/l)	33.07 (16.05–70.13)	20.5 (8.08–43.97)	2.742	0.006*
RF (IU/l)	164.0 (66–209)	90.5 (22.5–176.5)	3.228	0.001*
DAS28	5.81 ± 1.15	5.22 ± 1.10	3.625	0.596
Sharp	68 (11–142)	11 (1–71.75)	4.461	< 0.0001*
HAQ	1.69 ± 0.60	0.88 ± 0.58	7.826	< 0.0001*
Joint function grade (I:II:III:IV)	1:36:40:9 (1.2%:41.9%:46.5%:10.5%)	13:77:19:0 (11.9%:70.6%:17.4%:0%)	39.473	< 0.0001*
Joint X-ray staging (I:II:III:IV)	9:12:29:36 (10.5%:14.0%:33.7%:41.9%)	32:18:34:25 (29.4%:16.5%:31.2%:22.9%)	13.964	0.003*

Mean and standard deviation for the continuous variables and number of participants (n) and percentages (%) for the categorical variables.

*P value < 0.05.

#### Comparison of disease activity, joint function, and joint structural damage among RA with different balance capacity

Joint swelling count, joint tenderness count, ESR, CRP, and RF in poor-balance group were higher than that in good-balance group, and the differences were statistically significant (*P* < 0.05), suggesting that the disease activity was higher in female RA with poor balance (Table 3). Component ratios of joint function grading (grades I–IV) were significant different among female RA with different balance capacity (*P* < 0.0001). The percentages of grades III and IV in poor-balance group were higher than those in good-balance group (46.5% vs. 17.4% and 10.5% vs. 0.0%). HAQ was significantly different among female RA with different balance capacity (*P* < 0.0001), and the median HAQ in poor-balance group was higher than that in good-balance group. Component ratios of X-ray staging (grades I–IV) were also significantly different among RA with different balance capacity (*P* = 0.003). The proportion of grades III and IV of X-ray in RA with poor balance was higher than those in RA with good balance (33.7% vs. 31.2% and 41.9% vs. 22.9%). The median Sharp score was higher in poor-balance group compared to good-balance group (*P* < 0.0001). The results suggest that joint damage was more severe in female RA with poor balance.

### Logistic regression analysis of VOPF in RA patients

With the occurrence of vertebral spinal OPF (0 = no OPF, 1 = OPF) as the dependent variables and age, BMI, DAS28, SMI, BBS, OP (0 = no OP, 1 = OP), GCs usage (0 = no GCs usage, 1 = GCs usage) as independent variables, we used binary logistic regression analysis (LR Backward) to determine potential risk factors for VOPF in female RA patients, and the results indicated that SMI (OR = 0.386, 95% CI 0.243–0.614, *P* < 0.0001) and BBS (OR = 0.952, 95% CI 0.929–0.976, *P* < 0.0001) were protective factors for VOPF in female RA; age (OR = 1.112, 95% CI 1.065–1.160, *P* < 0.0001), OP (OR = 10.137, 95% CI 4.224–24.330, *P* < 0.0001) and GCs usage (OR = 3.532, 95% CI 1.427–8.741, *P* = 0.006) were risk factors for VOPF in female RA (Table 4).

**Table 4:** Logistic regression analysis of VOPF in female RA.

Dependent variables	Independent variables	B	Wald	*P*	*OR*	95% CI
VOPF (0 = no OPF, 1 = OPF)	Age	0.106	23.719	< 0.0001*	1.112	1.065–1.160
	SMI	− 0.951	16.188	< 0.0001*	0.386	0.243–0.614
	BBS	− 0.049	14.627	< 0.0001*	0.952	0.929–0.976
	OP	02.316	26.886	< 0.0001*	10.137	4.224–24.330
	GCs usage	1.262	7.450	0.006*	3.532	1.427–8.741

*P value < 0.05.

## Discussion

As a chronic systemic inflammatory disease, RA closely associated with local and systemic osteoporosis, and for some patients, local bone loss was already present in the preclinical phase of RA^22^. We demonstrated that female RA patients had higher incidence of OP and VOPF compared to controls. Tong JJ et al. proposed that OP in RA was closely associated with high age, female gender, low body mass index, and high disease activity, while OPF was associated with high age and glucocorticoid use^23^. Raterman et al. proposed that OPF was associated with sarcopenia, inflammatory status of RA itself, and glucocorticoid use^24^, and RA patients who had longer disease duration and more difficult to control inflammation are at higher risk of developing OPF (i.e., hip and vertebral fractures). The prognosis of RA patients with sarcopenia was relatively worse, with a significantly higher rate of reduced BMD, falls, and fractures. The catastrophic situation usually caused a high disease burden, undermined the patient's quality of life, and increased health care costs and mortality.

Compared to healthy individuals, RA patients had a higher rate of sarcopenia. Sarcopenia was defined as the loss of skeletal muscle mass and strength, both of which gradually declined with age, and it was reported that a prevalence of sarcopenia of 5–13% in older adults aged 60–70 years^25^. In contrast, the European Working Group on Sarcopenia in the Elderly (EWGSOP) showed a prevalence of up to 29% in elderly people in the regular community^26^. Previous studies showed that the prevalence of sarcopenia in RA patients was 29.6–43.3%^27–31^, which was significantly higher than normal people. Thus, in our study, the incidence of sarcopenia in female RA patients was 60.0%, which might indicated that sarcopenia were more frequent in women. As in one previous study reported, female gender was a risk factor for sarcopenia in RA patients.

Mie Torii et al. found that sarcopenia was positively correlated with the patient's age, disease duration, joint destruction and degree of malnutrition, and negatively correlated with the use of bDMARDs^27^. In a study of Takeshi Mochizuki et al., the age, BMI, CRP and hip BMD of RA were associated with sarcopenia^29^. Ange Ngeuleu et al. studied 123 RA patients and found that the occurrence of sarcopenia was associated with DAS28, ESR, bone erosion, increased cardiometabolic risk and HAQ^30^. Li et al. found that disease duration influenced development of sarcopenia, DAS28 and HAQ predict occurrence^31^. In present study, female RA patients with sarcopenia were usually older, had a longer disease duration and lower BMI than those without sarcopenia, which was consistent with the results of previous studies. We also confirmed that GCs usage was more often in sarcopenia group and this indicated that sarcopenia was correlated with GCs. ESR and CRP were generally higher in female RA with sarcopenia, suggesting that disease activity was higher in the sarcopenic group, so sarcopenia was closely related to disease activity in RA itself. We also found that female RA with sarcopenia had a significantly higher proportion of grades III and IV of joint X-ray, higher Sharp scores, and higher HAQ scores, suggesting that female RA with sarcopenia had more joint involvement and more severe bone destruction.

Poor balance was common in elder people, and it always predicted multiple complications such as falls, fractures, brain injury, disability and death. Urrunaga-Pastor et al. showed that the factors associated to the presence of poor balance ability were: alcohol consumption, having suffered at least one fall in the last year, exhaustion and at having at least one comorbidity^32^. In previous study, Chen et al. studied 238 RA patients and found that RA patients with poor balance often had higher disease activity, severer structural damage, worse joint function and a higher incidence of VOPF compared with RA patients with good balance^33^. We got similar conclusions. Our study suggested that age, disease duration, GCs usage, JSC, JTC, ESR, CRP, and RF were generally higher in poor-balance group compared to that in good-balance group. There was an increasing trend about percentages of grade III and IV of joint function along with poor balance in female RA, while HAQ scores also elevated. Furthermore, the percentages of grade III and IV of joint X-rays and sharp scores in poor-balance group were higher than that in good-balance group. All of above suggested female RA with poor balance always had higher disease activity, more severe structural damage, and worsen joint function.

There were few studies about relationships between balance ability, sarcopenia and VOPF in female RA patients. In our study, we showed a higher prevalence of sarcopenia and poor balance found in female RA patients with VOPF. Sarcopenia could lead to a decrease in muscle strength, neuromuscular weakness, and balance disorders due to immobility. Previous studies had shown that both sarcopenia and poor balance were significantly associated with an increased risk of falls^34^. We found that the incidence of OP was significantly higher in female RA with sarcopenia or poor balance, and on top of this, falls were highly likely to lead to VOPF if they occurred.

In the present study, we analyzed the factors influenced the occurrence of VOPF in female RA patients by binary logistic regression analysis (LR backwald), and the results suggested that SMI (OR = 0.386, 95% CI 0.243–0.614, *P* < 0.0001) and BBS (OR = 0.952, 95% CI 0.929–0.976, *P* < 0.0001) were protective factors while age (OR = 1.112, 95% CI 1.065–1.160, *P* < 0.0001), OP (OR = 10.137, 95% CI 4.224–24.330, *P* < 0.0001) and GCs usage (OR = 3.532, 95% CI 1.427–8.741, *P* = 0.006) were risk factors. This suggested that sarcopenia, poor balance, OP and GCs usage were closely related to the occurrence of VOPF in female RA patients, with age increased, the more likely VOPF would occur; RA patients with both sarcopenia and poor balance were at increased risk of mobility, falls, fractures, and even death. This result suggested that it was necessary to pay attention to the health education of RA patients, strengthen their muscle strength training, improved their balance ability, and effectively avoided falls that lead to VOPF in routine clinical treatment to improve the quality of life of patients and avoid the deterioration of their symptom.

RA was mainly driven by augmented cytokine secretion, including TNF-α, Interleukin-6 (IL-6), and Interleukin-1 (IL-1). These cytokines could directly and indirectly activate osteoclasts, inducing bone loss, which could occur in the early stage of RA^22^. Moreover, inflammatory cytokines could halt osteoblast differentiation. In addition, inflammation could lead to osteoporosis through the systemic and local release of proteinases (metalloproteinases) that could directly degrade bone tissue. Other independent factors associated with bone destruction in RA were autoantibodies against citrullinated proteins (ACPA)^35^. ACPA positivity was related, in a titer-dependent manner, to systemic OP^36^, even before the clinical onset of RA. Compared to healthy individuals, skeletal muscle cell mass was reduced in most RA patients while fat mass was maintained or increased, paticularly in women. In clinical aspect, besides advanced age, sarcopenia had been notoriously associated with long-term inflammatory condition^37^. Visser et al. had reported that high levels of TNF-α, IL-6, and CRP in the blood of RA patients were associated with decreased muscle strength, and these cytokines might also increase protein degradation during muscle tissue synthesis^38^. According to the study of Dalle et al., IL-1 also palyed an important role in sarcopenia in RA^39^. In addition, rheumatoid musculoskeletal symptoms including pain, swelling and stiffness preclude patients from exercise and physical activity, leading to disuse atrophy of the skeletal muscles^40^, resulting in an imbalance between protein metabolism and catabolism; the persistent inflammation exhausted myocytes and impeded muscle growth; the nutritional deficit resulted from disuse and flammation further aggravated the impasse^41^.

Long-term exposure to inflammation in RA severely affected both bone and muscle mass. In addition to sarcopenia, poor balance might also be a risk factor for VOPF. If without timely intervention, the probability of VOPF was greatly increased. TNF-α and IL-6 that were used in RA treatment could be an alternative treatment option for sarcopenia^42^. Metsios et al. who evaluated BC, ESR, CRP levels, and DAS28 scores before and after 12-week anti-TNF therapy showed that no significant change occurred, whereas marked improvement was achieved in disease activity with this treatment in 20 patients with RA^43^. Therefore, if we apply bDMARDS, which antagonizes TNF-α and IL-6, to inhibit inflammatory cytokine-induced proteolytic metabolism in a timely manner at an early stage of the disease, we can prevent the development of sarcopenia and OP. Otherwise, it was important to improve the balance ability of RA patients to prevent fractures due to falls by following appropriate instructions, guiding patients to exercise properly and strengthening skeletal muscle strength.

Our study had some limitations. First of all, the number of patients were small. Furthermore, in our study, only female RA and controls were enrolled. Sarcopenia in male patients with RA was not examined. Also a discrepancy in the demographic and clinical characteristics might affect the results. This is another limitation of our study.

In conclusion, RA patients are susceptible to VOPF, especially females, which could be influenced by various factors. This study clearly showed that sarcopenia and poor balance were risk factors for VOPF, and they had synergistic effects on the occurrence of VOPF in female RA. Sarcopenia and poor balance were closely related to the disease activity and joint structural damage in RA. In our clinical work, we should pay attention to the muscle quality, strength, and balance ability of RA, while actively monitor and control the disease activity at an early stage to minimize the occurrence of VOPF.
